# Fabrication of Intragastric Floating, Controlled Release 3D Printed Theophylline Tablets Using Hot-Melt Extrusion and Fused Deposition Modeling

**DOI:** 10.3390/pharmaceutics12010077

**Published:** 2020-01-17

**Authors:** Bhupendra Raj Giri, Eon Soo Song, Jaewook Kwon, Ju-Hyun Lee, Jun-Bom Park, Dong Wuk Kim

**Affiliations:** 1 College of Pharmacy & Research Institute of Pharmaceutical Sciences, Kyungpook National University, Daegu 41566, Korea; giribhupen77@gmail.com (B.R.G.); djstn0424@naver.com (E.S.S.); kjw11156@naver.com (J.K.); 2 College of Pharmacy, Sahmyook University, Seoul 01795, Korea; jelly_3004@naver.com (J.-H.L.); junji4@gmail.com (J.-B.P.)

**Keywords:** theophylline, hot-melt extrusion, fused deposition modeling 3D printing, gastro-retentive floating system, dissolution kinetics, controlled release

## Abstract

This work presents a novel approach for producing gastro-retentive floating tablets (GRFT) by coupling hot-melt extrusion (HME) and fused deposition three-dimensional printing (3DP). Filaments containing theophylline (THEO) within a hydroxypropyl cellulose (HPC) matrix were prepared using HME. 3DP tablets with different infill percentages and shell thickness were developed and evaluated to determine their drug content, floating behavior, dissolution, and physicochemical properties. The dissolution studies revealed a relationship between the infill percentage/shell thickness and the drug release behavior of the 3DP tablets. All the developed GRFTs possessed the ability to float for 10 h and exhibited zero-order release kinetics. The drug release could be described by the Peppas–Sahlin model, as a combination of Fickian diffusion and swelling mechanism. Drug crystallinity was found unaltered throughout the process. 3DP coupled with HME, could be an effective blueprint to produce controlled-release GRFTs, providing the advantage of simplicity and versatility compared to the conventional methods.

## 1. Introduction

The limitations of orthodox oral drug delivery systems such as fast emptying time and incomplete absorption due to physiological variations, particularly unpredictable gastrointestinal (GI) transit and emptying times as well as metabolic degradation in the lower GI tract, resulting in inferior pharmacokinetics with therapeutic failure for a few drugs [1]. This scenario has led to a scientific undertaking to identify better alternatives. The development of gastro-retentive drug delivery systems (GRDDS) was brought into the limelight in order to overcome the above constraints by retaining drugs in the stomach for an extended period, ensuring optimal bioavailability. Numerous strategies have been explored for GRDDS to slow the gastric emptying rate; for example, intra-gastric floating systems [2,3], bio-adhesives [4], swelling and expanding [5], and low/high-density systems [6]. The gastro-retentive floating tablet (GRFT) is a low-density system with enough buoyancy to remain afloat above the gastric content in the stomach for a prolonged and pre-determined period of time without interference from the normal peristalsis of the GI tract.

In general, GRFTs are categorized into effervescent and non-effervescent systems [7]. In the effervescent system, CO_2_ air bubbles get liberated and entrapped into swollen hydrocolloids by the chemical reaction between effervescent agents such as magnesium carbonate, sodium bicarbonate, calcium carbonate, citric acid, etc., with gastric contents of the stomach that provides enough buoyancy for floating. Gas needs to be liberated and entrapped first; therefore, this system shows floating after a lag time, resulting in the risk of premature emptying of the dosage form from the stomach. In addition, unlike the swelling and mucoadhesive systems, the risk of gastric injuries, and unwanted side effects are low for floating devices [8]. On the other hand, the non-effervescent system uses a high percentage (20–75% *w*/*w*) of one or more gel-forming, highly swellable polymers into the tablet or capsule matrix to gain adequate buoyancy [7]. As a result, the drug release profile of dosage forms cannot be altered without adjusting the floating property and vice versa. In general, the conventional gastro-retentive systems always possess threats of floating lag time, premature gastric emptying, variable gastric retention time, and inconsistent drug release rate [9]. Therefore, to address the above challenges, a need is felt to develop a GRFTs system exploiting the novel 3DP and HME technologies that could stay afloat and releases the drug in a controlled manner.

3DP, also known as ‘additive manufacturing’ and ‘rapid prototyping’, is a manufacturing process used to create 3D physical objects by depositing materials in consecutive layers in the *x*, *y*, and *z*-axes with the aid of digital sketches obtained from a computer-aided design (CAD) software. Current 3DP technologies include (i) particle fusion-based methods, such as selective laser sintering (SLS), (ii) stereolithography (SLA), (iii) inkjet printing, and (iv) extrusion-based methods such as direct ink writing and fused deposition modeling (FDM). Among these, FDM is one of the most utilized and commercialized techniques for pharmaceutical applications due to its easy accessibility, efficiency, and cost-effectiveness, as well as its compatibility with a range of pharmaceutical thermoplastic polymers [10,11,12,13]. The starting material for FDM is produced via HME by blending the active pharmaceutical ingredients (APIs) with thermoplastic polymers to produce long, cylindrical, rod-shaped filaments. These filaments are fed into the heating nozzle, melted, and deposited layer-by-layer with FDM to produce the desired 3D shapes. The coupling of FDM and HME in one-unit operation has great potential for producing personalized dosage forms in-house for instant consumption. Thus, the combination is considered a revolutionary change in the context of pharmaceutical manufacturing [14].

More recently, a few studies have exploited novel 3DP technique to prolong gastric retention time of curcumin [15], acyclovir [16], amoxicillin [17], domperidone [18], riboflavin [19], and propranolol hydrochloride [20] however, the underlying drug release mechanism of 3D printed theophylline floating tablets remains unclear. Therefore, in this study, we aim to (i) develop controlled release THEO hollow tablets as intragastric floating devices by employing 3DP coupled with HME technology, and (ii) investigate the underlying drug release phenomenon from the 3DP THEO-HPC matrix tablets based on the mathematical modeling equations. The 3DP tablets with varying shell thickness and infill percentages were designed using 3D CAD software, and tablets were developed through an FDM 3D printer. Pharmaceutical grade hydroxypropyl cellulose (HPC) filaments need to be produced in-house with HME as no commercial HPC filaments are available. All the prepared 3DP GRFTs tablets could afloat for 10 h without showing any floating lag phase. Utilizing this approach could reduce the frequency of administration, minimize fluctuations in the plasma drug concentration with constant and steady drug release, and improve the overall therapeutic efficacy of THEO, leading to improved patient compliance and quality of life of patients. However, the in vivo analysis of the prepared dosage forms will be investigated in our future studies. To the best of our knowledge, this is the first work to exploit FDM 3DP paired with HME technology to develop a controlled release 3DP THEO-loaded HPC floating tablets.

## 2. Material and Methods

### 2.1. Materials

THEO was purchased from the Tokyo Chemical Industry Co. (Tokyo, Japan). HPC was donated by Hanmi Pharmaceutical Co. (Hwasung, Korea). Stearic acid (SA) was supplied by JUNSEI Chemical Co. (Tokyo, Japan) and was used as a plasticizer. All the other chemicals were of reagent grade and were used without further purification.

### 2.2. Preparation of THEO-Loaded Filaments

THEO, HPC, and SA were evenly mixed at a weight ratio of 30:70:7 using a bench-top blender. The mixture was fed and extruded using a twin-screw extruder (Process 11, Thermo Fisher Scientific, Karlsruhe, Germany). The mixture was extruded at 150 °C for all zones with a standard screw configuration at a screw speed of 50 rpm. A 1.2-mm diameter rod-shaped die was used to prepare filaments. The brittleness and flexibility of the produced filaments were examined manually, and the filament diameter was measured in 5–10 cm intervals with a digital Vernier caliper.

### 2.3. Fabrication of FDM 3DP Tablets

Seven 3DP tablets (T1–T7) with different infill densities and shell thickness were produced using drug-loaded filaments via a standard FDM 3D printer (ANET-A8; Shenzhen Anet Technology Co. Ltd., Shenzhen, China). The 3DP tablets were created using the browser-based 3D design tool Tinkercad (Autodesk Inc., San Rafael, CA, USA), and the designed templates were exported as stereolithography (.stl) file into Cura v. 15.04.04 (Ultimaker B.V., Geldermalsen, The Netherlands). The selected geometry for the dosage form was a flat-faced cylindrical tablet with the dimensions X = 10.0 mm, Y = 10.0 mm, and Z = 5.0 mm. Cylinder-shaped tablets with different infill densities (0%, 10%, 20%, and 30%) and shell thickness (0, 0.4, 0.8, and 1.2 mm) (Table 1) were prepared to investigate the floating capability of the tablets. Other printer settings were as follows: standard resolution with the raft option deactivated and an extrusion temperature of 210 °C, speed of 20 mm/s while extruding, speed of 90 mm/s while traveling, and a layer height of 0.10 mm.

### 2.4. Physicochemical Characterization

#### 2.4.1. Determination of Drug Loading in 3DP Tablets

In order to determine the THEO content, each tablet or filament was cut into small pieces, weighed (approx. 0.1 g), and placed in a 100 mL volumetric flask containing methanol under magnetic stirring until complete dissolution to ensure complete drug release. Then the solution was diluted (100-fold), and the concentration of THEO was measured at UV 270 nm (*n* = 3).

#### 2.4.2. Scanning Electron Microscopy (SEM)

The shape and surface morphology of the drug-loaded filaments and 3DP tablets were examined using SEM (SU8220; Hitachi; Tokyo, Japan) operating at an accelerated voltage of 5.0 kV. Samples were affixed onto a brass specimen holder using double-sided adhesive tape, and the samples were made electrically conductive by coating with platinum (6 nm/min) in a vacuum (0.8 Pa) for 4 min at 15 mA using an EmiTeck Sputter Coater (K575 K).

#### 2.4.3. Differential Scanning Calorimetry (DSC)

A differential scanning calorimeter (TA DSC Q20; TA Instruments; Newcastle, DE, USA) was employed for thermal analysis. Approximately 5 mg of samples were weighed, sealed, and placed in an aluminum pan. The samples were heated from 50 °C to 300 °C at a constant temperature change rate of 10 °C/min under a nitrogen gas flow of 50 mL/min. All the measurement data were analyzed using the TA 2000 analysis software.

#### 2.4.4. Powder X-Ray Diffraction (PXRD)

In order to evaluate the physical form of all the powder samples, a powder X-ray diffractometer (D/MAX-2500; Rigaku; Tokyo, Japan) equipped with a copper anode operated using Cu Kα radiation (1.54178 Å, 40 kV, and 40 mA). Samples were scanned from 5° to 50° using the step scan mode with a step size of 0.05°/s at room temperature and a 2θ diffraction angle to obtain the diffraction patterns.

### 2.5. In Vitro Dissolution and Floating Study

The floating behavior of the 3DP tablets was evaluated by placing the tablets in a transparent glass beaker containing 100 mL of 0.1 N HCl (pH 1.2) on a magnetic stirring at 50 rpm at room temperature. Furthermore, an in vitro drug release study was performed for all 3DP tablets using a USP type II dissolution apparatus (DT 620; ERWEKA; Heusenstamm, Germany). The tablets were placed in 900 mL of 0.1 M HCl medium maintained at 37 ± 0.5 °C and agitated at 50 rpm. Samples (2 mL) were extracted at predetermined intervals, and an equal amount of fresh media was immediately replenished to compensate for the loss during sampling. Prior to analysis, the collected samples were filtered with a PTFE membrane syringe filter Ø 0.45 µm and analyzed with a UV–vis spectrometer (UV-1800; Shimadzu, Kyoto, Japan) at 270 nm.

### 2.6. Dissolution Kinetics Studies

One of the primary goals of this work was the formulation of zero-order kinetics THEO loaded 3D tablets and understood the drug release mechanism of the gastro-retentive 3D dosage forms. Thus, various mathematical models such as Ritger–Peppes, Peppes–Sahlin, and zero-order release models were employed, and the correlation coefficient (*R*^2^) was calculated. Dissolution data analysis was conducted by comparing the dissolution profiles while statistically applying the mathematical models to quantify and characterize the drug release from the 3D tablets.

## 3. Results and Discussion

### 3.1. Preparation of Drug-Loaded THEO Filaments

Material choice is a key aspect factor in FDM 3DP. Most of the pharmaceutical grade polymers used in the preparation of conventional oral dosage forms cannot be extruded with HME into the desired filaments for printing. In addition, only a few studies investigating cellulose and its derivatives for 3DP applications are available [21,22,23]. Hydroxypropyl cellulose (HPC), chemically known as cellulose 2-hydroxypropyl ether, is generally used as a thickening agent, tablet binder, film-coating, and extended release-matrix former in oral dosage forms [24]. It is a non-ionic, water-soluble, and pH-independent polymer, commercially available in a few grades with different viscosities and a molecular weight ranging from 50,000–1,250,000 g·mol^−1^ [25]. The low glass transition (*T*_g_) temperature of HPC makes it pliable and easy to extrude through HME [26].

An HME-equipped with co-rotating twin-screw was used to extrude the mixtures of drug and cellulose polymer with a plasticizer to form long rod-shaped filaments. HME was carried out at 150 °C with a torque of 5–12 N/cm. The extruded HPC filaments loaded with THEO had a solid white appearance. Filaments with a consistent diameter in the range of 1.47 ± 0.01 mm and sufficient mechanical properties viz. strengths and flexibility suitable for FDM 3DP were produced with an HME die diameter of 1.2 mm. This increment in filament diameter compared to the HME die diameter (Ø 1.2 mm) is commonly known as the “die swell,” corresponding to the effect of the heat and high shear stress generated during HME processing [27]. The HPC was subjected to a slightly higher temperature (150 °C) than its lower *T*_g_ value (105 °C) as well as high shear stress during mixing between the continuously rotating twin-screw and the wall of the barrel. Upon leaving the die, the polymer chains try to recover from the deformation applied by the co-rotating screw by “relaxing” and increasing their radius of gyration, resulting in the expansion of the filament diameter [27].

### 3.2. 3DP of THEO Dosage Forms

Cylindrical, hollow tablets with varying shell thickness and infill percentages were successfully printed via 3DP. The outer shell of the tablets kept the inner portion hollow (replaced with air) to ensure the tablets remained in a low-density state. The mechanical properties of the devices were found to be satisfactory, and the devices were not friable and were easy to handle. As presented in Table 1, the tablet weight was found to depend on both the shell thickness and infill percentage, with the former having a greater influence. As reported in previous literature, 3DP tablets have a plastic-like aspect with an incredibly high tablet strength, which is difficult to quantify with a conventional tablet hardness tester [28]. The friability of all the formulations was found to be zero, and it was difficult to separate the layers by applying force with sharp surfaces or human nails, without cutting the layers.

It is important to note that the 3DP temperature is significantly higher (210 °C) than the HME temperature (150 °C). This is due to the different heating rates of the two processes. During the preparation of filaments, the processing temperature is maintained for 5 min or longer. On the other hand, for successful printing, the filament needs to be in a semi-solid state, and therefore, the temperature is higher at the printer nozzle. The filament passes through the hot nozzle of the printer for a brief period at a much faster speed and, therefore, experiences the heat for a much shorter duration than during HME. The high temperature at the nozzle turns the filaments into a semi-solid state, which is followed by rapid fusion and quick solidification at room temperature. To attain such a rapid state change (from semi-solid to solid), the printers’ temperature is usually elevated to around 210–250 °C. Interestingly, THEO-loaded HPC filaments were found to withstand this rise in temperature. The higher melting point of THEO (273 °C) compared to the 3D printer setting temperature (210 °C) allows the consistent flow of the semi-solid state filament from the printer’s nozzle, and the low room temperature (25 °C) causes rapid solidification of the printed structure. However, lowering the nozzle temperature below 200 °C was found to increase the filament viscosity and cause poor material flow from the nozzle, resulting in the blockage of the printer nozzle, and finally, termination of the printing process.

### 3.3. Physicochemical State Characterization

#### 3.3.1. Determination of Drug Loading

The chemical integrity of the drug in the 3DP tablets and filaments was analysed using a UV–vis spectrophotometer. Drug loading for the filaments was 302.83 ± 6.71 μg/mL (theoretical loading—300 μg/mL) and that for the printed tablets was 300.21 ± 1.49 μg/mL (theoretical amount—300 μg/mL), indicating no significant drug loss occurred during filament processing and tablet preparation. All the extruded filaments obtained in the present study showed appropriate characteristics for 3DP in terms of diameter, strength, flexibility, and brittleness.

#### 3.3.2. Scanning Electron Microscopy (SEM)

**Figure 1:**
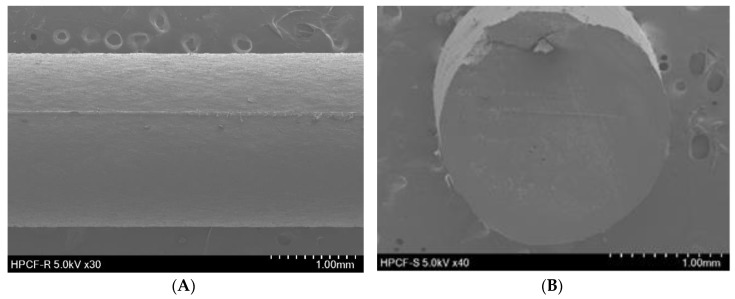
SEM images of hot-melt extruded filament. (**A**) Exterior appearance (30×) and (**B**) cross sectional shape (40×).

**Figure 2:**
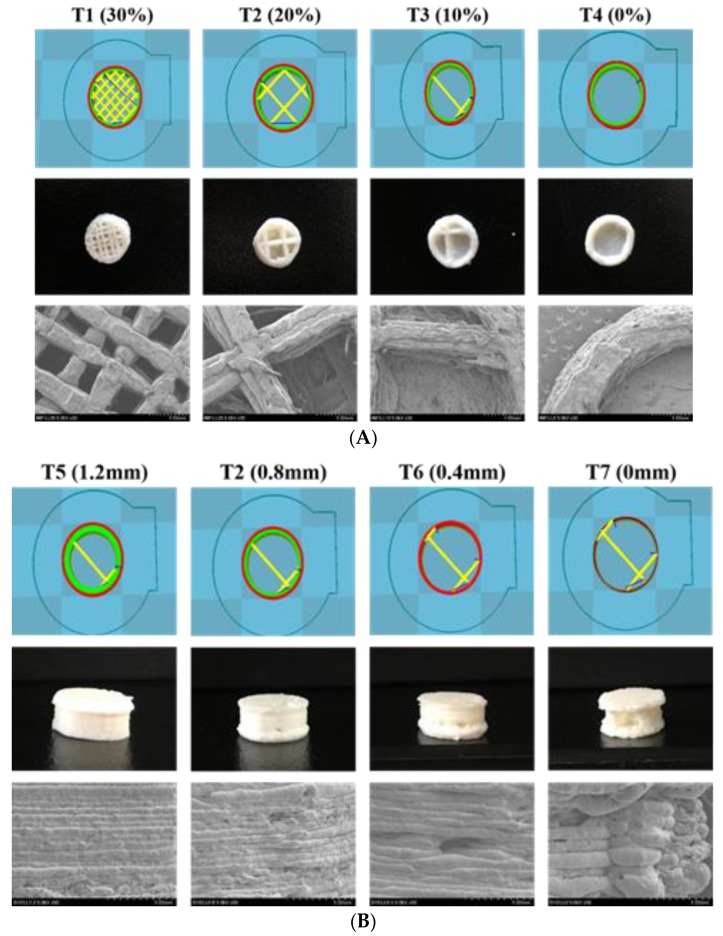
3D tablet design templates, tablet photographs, and SEM images of 3D printed tablet surface with (**A**) constant shell thickness (0.8 mm), different infill percentages (T1 30%, T2 20%, T3 10%, and T4 0%) and (**B**) constant infill percentage (20%), different shell thickness (T5 1.2 mm, T2 0.8 mm, T6 0.4 mm, and T7 0 mm).

SEM was used to investigate the topography of the extruded filament and the 3DP tablet structure. As depicted in Figure 1, the hot-melt extruded filament had a rod-shaped, robust, homogeneous smooth surface without a porous structure, indicating the absence of air pores and cavities in the filament. Figure 2A shows the pictures and SEM images of the 3DP THEO tablets with different infill percentages. The tablet with the highest infill density (T1, 30% infill) contained numerous small holes with a dense mesh-like structure, whereas the structure appeared loose and contained larger air cavities as the infill percentages decreased (T2 20%, T3 10%, and a single large cavity was found in T4 with 0% infill). Figure 2B shows the layer-layer deposition structure of the printed tablet, suggesting the influence of shell thickness. As can be observed from the 3DP tablet pictures and SEM images, the tablet with high shell thickness (T5) had a compact rigid structure with a slightly smooth outer surface. However, upon reducing the shell thickness (from T5, 1.2 mm to T7, 0 mm), a loose gap appeared between the successive layers with increasing layer thickness, resulting in a coarse and rough outer surface.

Overall, all the prepared 3DP tablets were observed to have slightly uneven and rough external surfaces, among which, T7 shows the maximum roughness (Figure 2). This might be correlated with the 3D printer resolution and shell thickness. In 3DP technology, the nozzle moves in three dimensions, i.e., horizontally (XY axis), and the build platform goes vertically downwards (Z-axis) as the process continues. The XY resolution determines the physical area (length and breadth) of the 3DP objects and is generally found consistent whereas, the Z resolution that controls the layer thickness or layer height is fairly inconsistent [29]. Due to the reasons, the microscopic imaging of 3DP objects shows uneven, asymmetrical, and rough surfaces. However, post-processing or extra finishing steps might make the surface look smoother [29]. Further, a judicious selection of the FDM 3D printer, i.e., with higher resolution capabilities and performs well in all the three dimensions (XY and Z axis), could improve the quality of the printed object [30].

#### 3.3.3. Differential Scanning Calorimetry (DSC)

**Figure 3:**
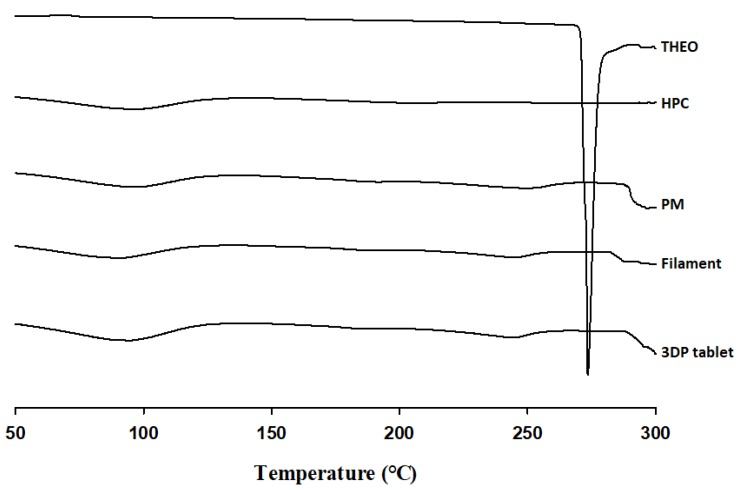
DSC curves of free THEO, hydroxypropyl cellulose (HPC), physical mixture (PM), THEO-HPC filament, and 3DP tablet. PM represents the physical mixture of THEO, HPC, and stearic acid (SA) at a 30:70:7 (*w*/*w*/*w*) ratio.

DSC was conducted to examine changes in the crystallinity of the bulk materials, the extruded filament, and the 3DP tablet during the thermal processes. As shown in Figure 3, THEO had a strong endothermic peak at approximately 275 °C, corresponding to its melting point. However, with the introduction of HPC, DSC thermograms were absent for the physical mixture (PM), filament, and the subsequent 3DP tablet. The absence of these peaks in the DSC thermograph may be due to the molecular dispersion of the THEO within the HPC polymer matrix, leading to reduced crystallinity that could not be detected with DSC. Therefore, PXRD was further employed for thermal analysis.

#### 3.3.4. Powder X-Ray Diffraction (PXRD)

**Figure 4:**
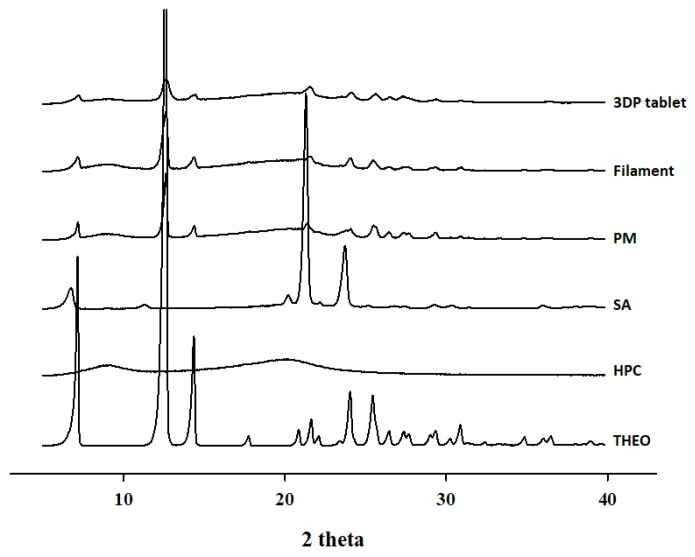
PXRD curves of 3DP tablet, THEO-HPC filament, PM, SA, HPC, and free THEO.

The use of high temperatures during HME and 3DP can lead to the degradation of thermolabile drugs and polymers. To further confirm the thermal properties of the samples, PXRD analysis was conducted. As illustrated in Figure 4, THEO showed numerous sharp diffraction peaks, with major peaks at 2θ = 7, 12, 14, and 24 that corresponded to the expected diffraction patterns [31], suggesting a highly crystalline nature. Surprisingly, the PXRD results for the PM, filament, and 3DP tablets were quite different from the results obtained with the DSC thermogram. The PM, filament, and 3DP tablets showed numerous peaks, all with reduced intensities. This may be due to the fact that the low-resolution DSC thermogram could not show crystallinity below 2% [32,33]. These observations suggest that the THEO crystal had converted to a partially crystalline state, with molecular dispersion of the drug into the polymer matrix. One possible explanation of this observation is the homogeneous mixing of the active material and the amorphous polymer under the thermal processing conditions for HME and FDM 3DP. However, in this work, no significant thermal degradation was observed for both the filaments and 3DP tablets.

### 3.4. In Vitro Floating and Dissolution Study

**Figure 5:**
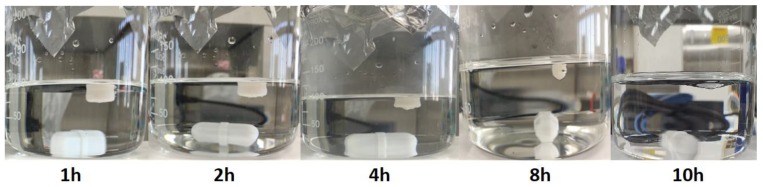
Photographs of 3DP tablet (T2) floating in dissolution medium (0.1 N HCl solution) at room temperature.

The floating ability was influenced by the density of the 3DP tablets, which in turn was found to be associated with the infill percentage of the tablets. The 3DP tablet (T2) with a density of approximately 0.79 g/cm^3^ was buoyant for more than 10 h (Figure 5), while the tablet with 60% infill sank to the bottom of the dissolution medium in less than 1 h (data not shown). Generally, tablets with lower infill percentages contain higher air content and, therefore, little to no floating lag time, owing to the low-density. In addition, the shell number did not show any marked differences in floating lag time for the 3DP tablets. Based on these observations, it can be inferred that the period of buoyancy for the 3DP tablets is closely related to their density, which in turn depends on the infill percentage.

**Figure 6:**
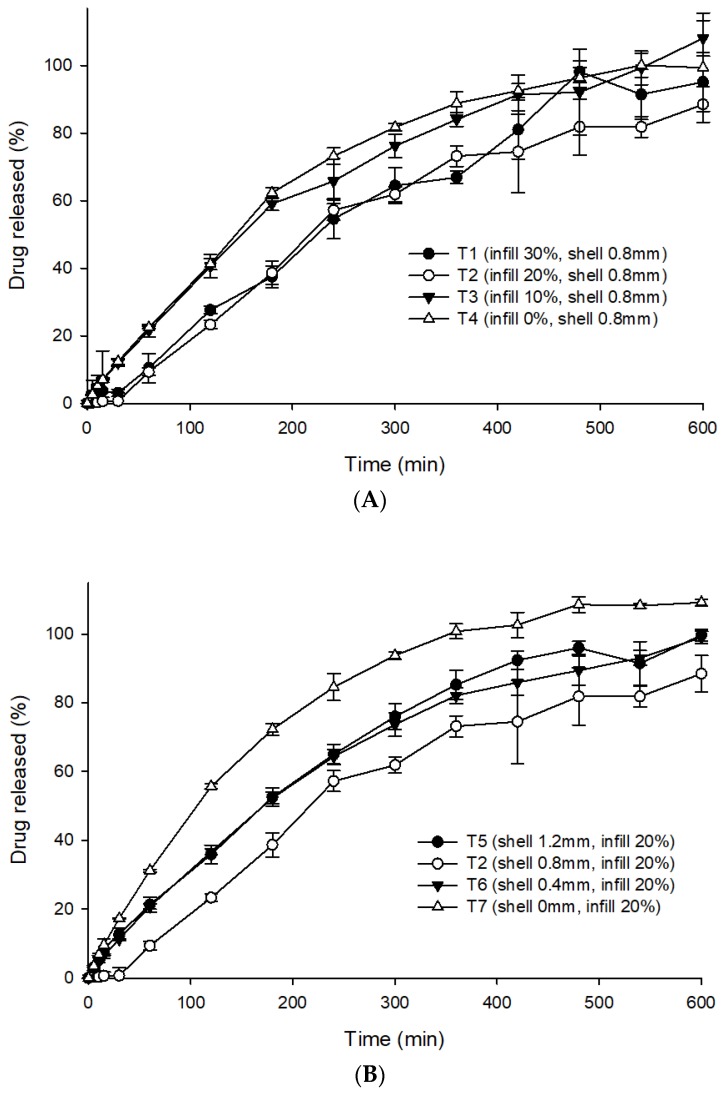
Drug release profiles of the 3DP tablets; (**A**) showing the influence of the infill percentages and (**B**) showing the influence of the shell thickness. Each value represents the mean ± standard deviation (*n* = 3).

In vitro dissolution studies for all seven formulations were performed in 0.1 N HCl (pH 1.2) to stimulate the gastric conditions of the stomach. In general, 0.1 N hydrochloric acid buffer (pH 1.2) is employed as the dissolution media to represent the acidic gastric fluid for gastro-retentive systems [16,18]. However, the dissolution test using a suitable biorelevant media, which seems to mimic the physiological in vivo dissolution better as compared to the buffer solutions may be worthy for future investigation [34]. The final run-out was over 90%, confirming that the drug was released at a relatively constant rate for 6 h before a plateau, with constant drug release thereafter (after 6 h). Figure 6A depicts the influence of infill percentage on the drug release profile of the 3DP THEO tablets. T1 (0.8 mm shell thickness and 30% infill) had a slow and extended drug profile compared with T4 (0.8 mm shell thickness and 0% infill). This could be mainly due to the large cavities/holes and large, loose gaps between the successive layers formed as the infill decreased. The higher porosity allows for quick penetration of the dissolution media into the tablet core, leading to rapid dissolution and diffusion of the drug from the THEO-HPC matrix. It has been reported that tablets with high infill percentages are harder and encounter a more intense retarding force that counteracts the positive effects of polymer dissolution, causing a delay in the drug release rate [35].

As illustrated in Figure 6B, the tablets with infill densities of 20% and varying shell thickness had different drug release rates. T2 (0.8 mm shell thickness, 20% infill), exhibited extended drug release rates because of its dense shell structure (as shown in SEM images, Figure 2B), while the tablets without shells (T7) exhibited fast drug release kinetics. This is mainly due to the thin and loose outer structure of T7, which dissociates promptly upon contact with the dissolution media, thus causing rapid drug release from the polymer matrix [35,36]. A large difference in drug release rate was observed with tablets having shell wall (T2, 0.8 mm) and without shell wall (T7, 0 mm); however, the difference was not that pronounced with tablets of different shell wall thickness (T2, T5, T6). Interestingly, the drug release profile of T5 (1.2 mm shell) was identical to the T6 (0.4 mm shell). Nevertheless, T2 (0.8 mm shell) displayed a slightly lower drug release rate compared to both T5 and T6, which might be possible due to the low printing resolution of the 3D printer (explained in Section 3.3.2).

Although the THEO 3DP tablets exceeded the tablet hardness limit on the hardness tester, the floating behavior and in vitro dissolution profile were found to be adequate, with 90% drug release over 8 h.

### 3.5. Dissolution Kinetic Studies

Mathematical models are essential tools for describing and quantitatively analyzing in vitro/in vivo drug release kinetics, predicting the release profile, and ensuring the optimal design of dosage forms [37]. In 1961, Higuchi developed the most recognized and commonly used kinetic equation to describe the release profile of drugs dispersed in homogeneous matrix systems [38]. There are a few assumptions with the Higuchi model [39,40]:

1. The initial drug concentration in a matrix system is much higher than the drug solubility.
2. Drug diffusion is one-dimensional because edge effects are insignificant.
3. The thickness of the dosage form is much larger than the size of the suspended drug particles (macro or nanoparticles).
4. The swelling or dissolution of the polymer carrier is negligible.
5. The drug diffusion coefficient is constant.
6. Perfect sink conditions are achieved in the release medium.

The Higuchi equation can only be used for an ideal controlled-release system because the first assumption provides the basis for the explanation of the pseudo-steady state [40,41]. Further, the equation considers the drug diffusivity to be constant, which is only valid for polymers that do not swell upon contact with the dissolution medium. Generally, with 3DP tablets, the tablet matrix may show multidimensional diffusion, and the matrix swelling phenomena cannot be neglected. Therefore, the Higuchi model is not relevant for this study.

Thus, to find the dissolution mechanism, the in vitro dissolution data were fitted in the Ritger–Peppas model (also known as the power law). The power law is a more comprehensive semi-empirical equation that describes drug release from polymeric systems when the drug release phenomenon is not known or if more than one type of mechanism is involved [42,43]. The equation is as follows:

(1) $$\frac{M_{t}}{M_{\infty }}=kt^{n},$$

where *M_t_* is the cumulative amount of drug dissolved over time *t*, *M_∞_* is the total amount of drug contained in a dosage form at the beginning of the release process, *k* is a constant incorporating the structural modifications and geometrical characteristics of the device, and *n* is the release exponent (related to the drug release mechanism) [44].

It is generally recommended to use the first 60% of the drug release curve for statistical analysis [35]. Based on the release parameter (coefficient of determination, R^2^), and the value of the release exponent (*n*), the mechanism by which the drug is released from the matrix system is proposed. For cylindrical tablets (Case I), 0.45 ≤ *n* corresponds to a Fickian diffusion where the drug molecules are released due to a diffusion process. The solvent transport rate or diffusion phenomenon is more dominant than the polymeric chain relaxation process. When *n* = 0.89, the model is non-Fickian (Case II transport), and the drug release corresponds to zero-order release kinetics. The drug release primarily involves swelling or relaxation of polymeric chains in the drug-loaded matrix system. Moreover, if 0.45 < *n* < 0.89, the model depicts non-Fickian or anomalous transport, and both diffusion and swelling mechanisms govern the drug release. At the end of Case II transport, a fast increase in the solvent absorption rate gives rise to the Super Case II transport model (when *n* > 0.89), during which the sorption process, tension, and breaking of the polymeric chains leading to drug release from the matrix system. Thus, the power law was applied to analyze the release profiles and the calculated data are listed in Table 2. T3 (*n* = 0.89, *R*^2^ = 0.98), T4 (*n* = 0.90, *R*^2^ = 0.99), T6 (*n* = 0.84, *R*^2^ = 0.98) can be considered to exhibit Case II transport. The drug release kinetics from these tablets may be governed by the swelling of the polymer matrix. In addition, T5 (*n* = 0.73, *R*^2^ = 0.99) and T7 (*n* = 0.79, *R*^2^ = 0.98) exhibit an anomalous transport, whereas T1 (*n* = 1.11, *R*^2^ = 0.98) and T2 (*n* = 1.39, *R*^2^ = 0.99) potentially exhibit Super Case II transport. Overall, the drug release exponent varied from 0.73–1.39, suggesting that both the matrix swelling and diffusion mechanism were involved in governing drug release kinetics from the prepared 3D tablet matrix.

To analyze the approximate contribution of these mechanisms (diffusional and relaxational), Peppas–Sahlin developed the following kinetics model [45]:

(2) $$\frac{M_{t}}{M_{\infty }}=k_{1}t^{m}+k_{2}t^{2m},$$

where *k*_1_, *k*_2_, and *m* are constants. The first term in the equation *k*_1_*t^m^* represents the Fickian diffusional contribution, whereas the second term *k*_2_*t*^2*m*^ represents the Case II swelling contribution. The coefficient *m* is a purely Fickian diffusional exponent. The amount of drug release due to the Fickian mechanism (*F*) is calculated as follows:

(3) $$F=\frac{1}{1+\frac{k_{2}}{k_{1}}t^{m}}$$

The ratio of both contributions can be calculated as:

(4) $$\frac{R}{F}=\frac{k_{2}t^{m}}{k_{1}}$$

**Figure 7:**
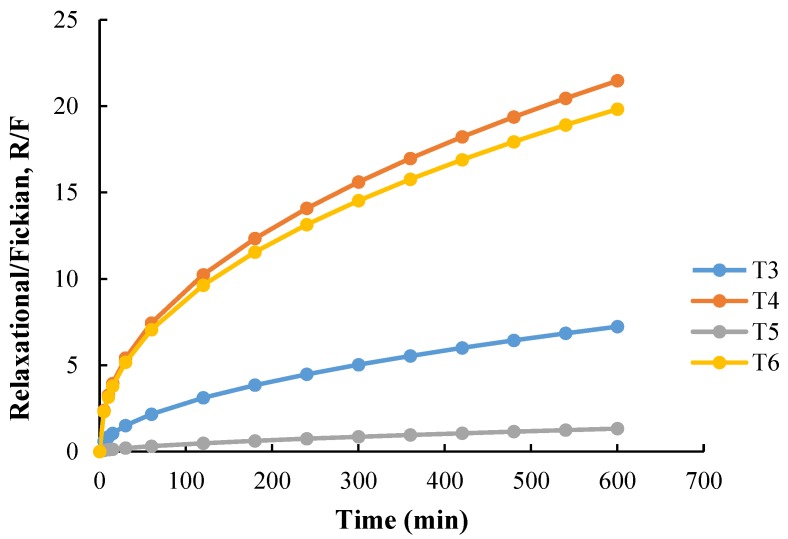
Swelling contribution (R) to the diffusion contribution (F), R/F ratio from T3, T4, T5, and T6.

Here, the diffusional contribution can be represented as a function of *t^m^*, and the relaxational contribution as *t*^2*m*^. The Peppas–Sahlin Equation (2) was applied to the dissolution profile of individually designed 3D tablets, and the ratio of relaxational contribution (*R*) over Fickian contribution (*R*/*F*) was calculated and plotted in Figure 7. Interestingly, though the correlation coefficients (*R*^2^) for T1, T2, and T7 were good (close to 1), the value for *k*_1_ was negative. A similar case was observed with the published report where the authors described the result as illogical and a possible consequence of the anomalous transport [35]. As shown in Figure 7, as the drug starts to dissolve in the T4 and T6, Fickian diffusion (*F*) decreases along with time, which indicates that the drug may be released as a result of polymer relaxation. The high mobility of carrier chains allowed easier solvent penetration into the tablet matrix, and the solvent diffusion rate was reduced below the relaxation rate. However, the *R*/*F* curve for T5 indicates that the influence of the swelling mechanism was significantly reduced due to the inclusion of Fickian diffusion in the drug release. Overall, the Peppas–Sahlin study revealed that both the swelling and diffusion mechanisms resulted in steady drug release from the 3DP tablets, and the swelling process had a relatively larger contribution during the entire drug release stage.

#### Zero-Order Drug Release

**Figure 8:**
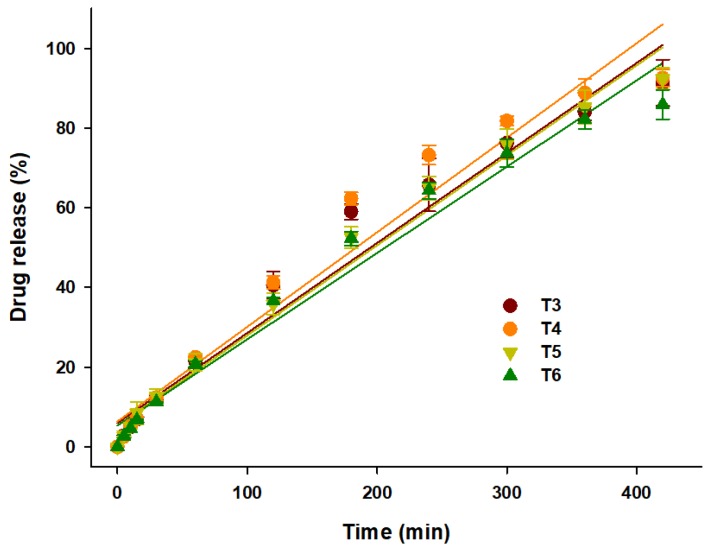
Linear fitting of drug release from T3, T4, T5, and T6 over 10 h.

Dosage forms that release the drug at a constant rate, resulting in a uniform drug plasma concentration, are desirable. Zero-order release can be represented by the equation:

(5) $$M_{t}=M_{o}+kt,$$

where *M_t_* is the cumulative amount of drug dissolved over time *t*, *M_o_* is the initial amount of drug (most of the time, *M_o_* = 0), and *k* is the zero-order release constant. As shown in the in vitro drug release curve (Figure 6), all seven formulations showed complete drug release within 10 h. The linear fitting of the in vitro drug release data is presented in Table 2. The linear regression (R^2^) values, which were close to 1, indicated that the prepared dosage forms showed a good fit for the zero-order. As predicted, T3 (*R*^2^ = 0.95), T4 (*R*^2^ = 0.94), T5 (*R*^2^ = 0.97), and T6 (*R*^2^ = 0.95) showed a steady and constant linear regression curve (Figure 8). The zero-order linear fitting results for T3, T4, T5, and T6 were consistent with the outcomes of the Ritger–Peppas and Peppas–Sahlin models. Hence, it can be concluded that the coupling of 3DP with HME could be an effective approach to develop zero-order release GRFTs.

## 4. Conclusions

THEO-loaded HPC filaments suitable for 3DP were successfully developed to produce 3DP intragastric floating tablets. The resulting 3D hollow tablets with different infill percentages and shell thickness showed sufficient buoyancy for approximately 10 h with zero-order drug release profiles, providing an alternative method to fabricate controlled-release intragastric floating drug delivery systems. Physical state characterization showed that the crystalline drug was molecularly dispersed within the cellulose matrix, resulting in reduced drug crystallinity. The infill density and shell thickness were found to be the key parameters for low-density, along with air entrapment in the inner core structure of the 3DP tablets, which altogether governs the in vitro drug release rate. Based on the Peppas–Sahlin model, both the swelling and diffusion phenomena were involved in the drug release process, with the former mechanism having a relatively larger contribution during the entire drug release stage. T3, T4, T5, and T6 had a constant and steady drug release profile with linear fitting, confirming that the drug release can be considered controlled (zero-order). Overall, this study demonstrates that the coupling of novel HME and FDM-based 3DP technologies could be an effective, efficient, and economical alternative to develop gastro-retentive dosage forms with better controlled-release rates as compared to the traditional pharmaceutical manufacturing. However, additional assessment for stability and in-vitro in-vivo correlation (IVIVC) studies of the 3DP floating formulations need to be further investigated. Despite considerable progress, 3DP technology is still in its early stages. Further innovations and development on product quality, novel structural design, better printing resolution, and proper regulatory mechanism, will facilitate this technology to become more practical in commercial manufacturing.

## Figures and Tables

**Table 1:** Physical properties of the 3D printed floating tablets with varied infill densities and outside shell thickness.

Formulation	Infill (%)	Shell (mm)	Diameter (X, mm)	Diameter (Y, mm)	Thickness (Z, mm)	Weight (mg)
T1	30	0.8	10.54 ± 0.15	10.30 ± 0.17	4.63 ± 0.02	353.71 ± 8.78
T2	20	0.8	10.22 ± 0.04	10.25 ± 0.01	5.02 ± 0.37	326.30 ± 17.63
T3	10	0.8	10.52 ± 0.11	10.12 ± 0.09	4.74 ± 0.13	297.30 ± 5.35
T4	0	0.8	10.48 ± 0.40	10.41 ± 0.27	4.68 ± 0.04	273.70 ± 4.72
T5	20	1.2	10.40 ± 0.25	10.57 ± 0.14	5.09 ± 0.03	401.96 ± 8.75
T6	20	0.4	10.55 ± 0.14	10.46 ± 0.36	5.13 ± 0.03	322.95 ± 5.32
T7	20	0	10.55 ± 0.13	10.40 ± 0.16	5.17 ± 0.11	266.24 ± 3.50

**Table 2:** In vitro dissolution parameters for the 3DP tablets.

Formulation	Ritger-Peppas Model		Linear Model		Peppas-Sahlin Model			
	Exponent, *n*	*R* ^2^	Slope, *k*	*R* ^2^	*k*_1_	*k*_2_	*m*	*R* ^2^
T1	1.11	0.9846	0.20	0.9876	−0.92	0.41	0.45	0.9814
T2	1.39	0.9927	0.19	0.9781	−0.55	0.13	0.56	0.9837
T3	0.89	0.9896	0.25	0.9519	0.80	0.20	0.52	1.0000
T4	0.90	0.9998	0.26	0.9422	0.87	0.01	0.73	0.9997
T5	0.73	0.9969	0.24	0.9708	1.20	0.02	0.63	0.9980
T6	0.84	0.9868	0.23	0.9594	0.40	0.45	0.44	0.9998
T7	0.79	0.9881	0.30	0.9043	−3.35	3.03	0.32	0.9894
